# Genome-Wide Identification and Characterization of the Potato bHLH Transcription Factor Family

**DOI:** 10.3390/genes9010054

**Published:** 2018-01-22

**Authors:** Ruoqiu Wang, Peng Zhao, Nana Kong, Ruize Lu, Yue Pei, Chenxi Huang, Haoli Ma, Qin Chen

**Affiliations:** State Key Laboratory of Crop Stress Biology for Arid Areas, College of Agronomy, Northwest A&F University, Yangling 712100, Shaanxi, China; wrq@nwafu.edu.cn (R.W.); zhaopeng@nwafu.edu.cn (P.Z.); knnnwafu@163.com (N.K.); Ruize@nwafu.edu.cn (R.L.); peiyue@nwafu.edu.cn (Y.P.); huangchenxiobst@nwafu.edu.cn (C.H.)

**Keywords:** genome-wide, potato, bHLH transcription factor, expression analysis

## Abstract

Plant basic/helix–loop–helix (bHLH) transcription factors participate in a number of biological processes, such as growth, development and abiotic stress responses. The bHLH family has been identified in many plants, and several bHLH transcription factors have been functionally characterized in *Arabidopsis*. However, no systematic identification of bHLH family members has been reported in potato (*Solanum tuberosum*). Here, 124 *StbHLH* genes were identified and named according to their chromosomal locations. The intron numbers varied from zero to seven. Most StbHLH proteins had the highly conserved intron phase 0, which accounted for 86.2% of the introns. According to the Neighbor-joining phylogenetic tree, 259 bHLH proteins acquired from *Arabidopsis* and potato were divided into 15 groups. All of the *StbHLH* genes were randomly distributed on 12 chromosomes, and 20 tandem duplicated genes and four pairs of duplicated gene segments were detected in the *StbHLH* family. The gene ontology (GO) analysis revealed that StbHLH mainly function in protein and DNA binding. Through the RNA-seq and quantitative real time PCR (qRT-PCR) analyses, *StbHLH* were found to be expressed in various tissues and to respond to abiotic stresses, including salt, drought and heat. *StbHLH1*, *41* and *60* were highly expressed in flower tissues, and were predicted to be involved in flower development by GO annotation. *StbHLH45* was highly expressed in salt, drought and heat stress, which suggested its important role in abiotic stress response. The results provide comprehensive information for further analyses of the molecular functions of the *StbHLH* gene family.

## 1. Introduction

Transcription factors are important in regulating gene expression on the transcriptional level. Transcription factors play essential roles in plant growth and development, and activate component syntheses and responses to environmental changes [1]. Most biological processes in eukaryotic cells or organisms are finely controlled by transcription factors on the transcriptional level.

Basic/helix–loop–helix (bHLH) transcription factors are widely found in animals and plants [2]. The bHLH superfamily is the second largest transcription factor family in plants [3]. Basic/helix–loop–helix transcription factors are named for their highly conserved alkaline/helix–loop–helix domains [4]. Transcription factors usually contain two different functional domains involved in DNA binding and protein interactions, which may be regulated by a variety of mechanisms, including the formation of dimers [5]. A bHLH transcription factor consists of two conserved motifs, a basic region and helix–loop–helix region (HLH region). The basic region is located at the N-terminal end, while the HLH region is located at the C-terminal end. The basic region contains ~15 amino acids of which six are basic amino acid residues. With DNA recognition and binding sites, the basic region is involved in DNA binding. The HLH region participates in dimerization [6,7] and is mainly composed of hydrophobic residues. The HLH region contains two amphipathic α-helices linked by a loop region with variable sequences [8,9]. Outside of the two conserved regions, the rest of the bHLH protein sequences are vastly divergent [10].

In yeast and other single cell eukaryotes, bHLHs are involved in chromosome separation and metabolic regulation processes [11]. In animals, bHLHs are mainly associated with sensing the external environment, cell cycle regulation and tissue differentiation [12,13,14,15]. The first bHLH protein reported in plants was Lc, which was encoded by the *R* gene. The Lc (L-myc) protein regulates the biosynthesis of flavonoid/anthocyanin in maize [16]. Basic/helix–loop–helix transcription factors are also involved in responding to light [17], cold [18] and hormone signals [19,20], regulating anthocyanin biosynthesis [21], epidermal cell fate determination [22], and in regulating the developmental patterns of roots [23] and flowers [24]. Recently, the overexpression of *bHLH30* led to upwardly curly leaves in *Arabidopsis* [25]. Furthermore, apple plants overexpressing the *MdbHLH104* gene possessed a high tolerance to iron deficiency [26].

Based on sequence homology and phylogenetic relationships, bHLH transcription factors are usually classified into six groups (from A to F) in animals [12,13]. These groups can be divided into several small subfamilies [27]. The six bHLH transcription factor groups diverge in function and binding elements [28]. Group A contains proteins such as MyoD and Twist and can bind to the E-box (CAGCTG). Group B includes many proteins with unrelated functions, such as Pho4 and R, which bind to the G-box (CACGTG). Group C contains bHLH transcription factors that have a second protein–protein interaction domain, for example, Per and Sim. The protein members can bind to core sequences of non-E-boxes (NACGTG/NGCGTG). Proteins of Group D contain the HLH region but lack the basic region. The representative proteins are Id, Emc and Heira. They can form heterodimers with typical bHLH proteins to function [29]. Group E consist of WRPW-bHLH proteins that have a Pro residue in the basic region instead of an Arg residue, such as Hairy and Enhancer of Split protein. Because of their low affinity for the E-box, Group E proteins bind to the N-box (CACGGC/CACGAC) prior to the E-box. Group F includes Collier/Olf1/EBF-bHLH (COE-bHLH) proteins that are vastly different from Group A–E proteins in sequence. These proteins have an additional domain that can function in DNA binding and dimerization [30]. However, research on bHLH proteins in plants is limited compared with in animals. In plants, the classification of bHLH is uncertain. Usually, it is divided into 15–26 groups [27,31]. Because of the identification of atypical bHLH proteins, the group number has risen to 32 [32].

With the availability of genome sequence data, more and more bHLH families have been identified and characterized in plant species. For example, a series of bHLH proteins have been identified in *Arabidopsis* [28], rice [33], cabbage [34], soybean [24], apple [35] and tomato [36]. However, the identification and analysis of bHLH proteins in potato (*Solanum tuberosum*) have been limited so far. A potato bHLH transcription factor that is the homolog of Petuniaan1 co-localized with the quantitative trait locus (QTL) on chromosome 9 [37]. There are also reports on bHLH co-factors interacting with other activators, such as AN1, MYBA1 and MYB113 [38,39]. Here, we performed a genome-wide analysis of the *bHLH* gene family in potato. The *bHLH* family members were identified using bioinformatics methods and a series of analyses of their characteristics, gene structures, gene ontology (GO) annotations, phylogenetic relationships and expression patterns were conducted. The study provides information necessary for further functional research on the *bHLH* family in potato.

## 2. Materials and Methods

### 2.1. Potato bHLH Sequence Retrieval and Analysis

The protein sequence data (DM_v3.4_pep_nonredundant) of potato were available at the Potato Genome Sequencing Consortium (PGSC) [40]. The Hidden Markov Model (HMM) was used to identify potato bHLH candidates, and the HMM profile of bHLH (PF00011) was downloaded from the Pfam [41] protein database. We used HMMER software [42] to search against the potato protein sequence data using default parameters. The locus IDs of acquired sequences were uploaded to PGSC to remove the non-representative transcripts. Then, the remaining sequences were checked for the conserved bHLH domain using CDD [43], Pfam [41] and SMART [44]. Finally, sequences with complete bHLH domains were preserved and named in order according to their locations on the chromosomes.

### 2.2. Gene Structure and Conserved Motif Characterization

The potato bHLH protein sequences were uploaded to ExPASy [45] to calculate the number of amino acids, molecular weights and isoelectric points. The intron numbers and chromosomal locations of potato *bHLH* genes were retrieved from the PGSC. The conserved motifs were predicted by the MEME program [46]. The parameters were set as any number of repetitions, optimum motif width of 10–200 residues, and searching for 20 motifs, with all other parameters in default. The predicated motif sequences were annotated by CDD [43], SMART [44] and InterPro [47]. The Gene Structure Display Server [48] was used to show the exon–intron structures of potato *bHLH* genes.

### 2.3. Chromosomal Location and Gene Duplication

The chromosome positional data for *bHLH* genes were retrieved from the PGSC genome browser. *bHLH* gene mapping was performed using MapChart software [49]. Gene duplication was confirmed using two criteria: (a) the shorter aligned sequence covered >70% of the longer sequence in length; (b) the similarity of aligned sequences was >70% [49,50]. Two genes located in the same chromosomal fragment of less than 100 kb and separated by five or fewer genes were identified as tandem duplicated genes [51]. The duplicated potato *bHLH* gene segments were confirmed by searching the Plant Genome Duplication Database [52].

### 2.4. Phylogenetic Analysis and Classification

The phylogenetic tree contained full-length amino acid sequences of bHLH from *Arabidopsis* [28] and potato (Table S1). All of the full-length amino acid sequences were aligned with ClustalX [53] using default parameters. MEGA6 [54] was used to generate the unrooted Neighbor-joining phylogenetic tree with the following parameters: 1000 times bootstrap test, Poisson model and pairwise deletions. The classifications of potato bHLH proteins were based on the topology and bootstrap values of the phylogenetic tree.

### 2.5. GO Annotation and RNA-Seq Data Analysis

The GO analysis of potato *bHLH* genes was performed using the Blast2GO program. The full-length amino acid sequences of potato bHLH proteins were uploaded to the program, and *Arabidopsis* was chosen as the reference database. The analysis contained three parts: molecular function, cellular component and biological process.

The RNA-seq data (DM_v4.03) [55] represented as fragments per kilobase per million (FPKM) values of *StbHLHs* in various tissues were downloaded from PGSC. The raw data of FPKM values were equalized by the mean of whole data. Subsequently, the processed data were transformed by log2. After then, HemI [56] was used to generate the expression heatmap of *StbHLH* genes.

### 2.6. Plant Materials, Growth Conditions and Stress Treatments

The potato cultivar *Desiree* was planted in a greenhouse of our laboratory at Northwest A&F University. Various tissues, including flower, stolon and mature tuber, were sampled [57]. There were three biological replicates for each sampled tissue.

A doubled monoploid potato variety (DM) with low levels of heterozygosity genome was used in the stress treatments. The plantlets were grown in Murashige and Skoog (MS) medium that contained 3% sucrose and 0.8% agar at pH 5.9, and sustained in an artificial climate chamber with a photoperiod of 16 h light/8 h dark at 22 ± 1 °C. The four-week-old plantlets were transferred into containers of 1/2 MS liquid medium and sustained for a week with the same growth conditions as before. The abiotic stress conditions were referred to previous reports [55]. The heat stress was conducted at 35 °C. For salt and drought stresses, the plantlets were treated with 150 mM NaCl and 260 mM mannitol, respectively. The aboveground parts of plantlets were collected at 0 and 24 h after treatment. All of the collected materials were immediately frozen in liquid nitrogen and stored at −80 °C.

### 2.7. RNA Extraction and qRT-PCR Analysis

An RNAsimple Total RNA Kit (BioTeke, Beijing, China) was used to extract the total RNA of the sampled plant materials. The cDNA was synthetized using a First Strand cDNA Synthesis Kit. The cDNA was diluted 10 times with nuclease-free water. All of the procedures were conducted according to the manufacturers’ protocols.

The specific potato *bHLH* gene primers for quantitative real-time PCR (qRT-PCR) were designed with Primer Premier 5 (Table S2). The qRT-PCR was conducted in a 20-μL reaction system with the following components: 10 μL KAPA SYBR FAST qPCR Kit Master Mix (2×) Universal, 1 μL 10 μM forward primer, 1 μL 10 μM reverse primer, 1 μL diluted cDNA and 7 μL ddH_2_O. A Bio-Rad CFX96 Real Time PCR System was used for the qRT-PCR. The procedure was: 95 °C for 2 min and 40 cycles of 95 °C for 5 s and 60 °C for 30 s. A melt curve was generated from 65 °C to 95 °C with increments of 0.5 °C every 5 s. Each gene was detected in three biological replicates and two technical replicates. The internal reference gene was *ef1α*. The relative expression was calculated using the 2^−ΔCT^ method for the tissue expression profile and 2^−ΔΔCT^ method for expression profile under stress [58].

## 3. Results and Discussion

### 3.1. Identification and Characterization of bHLH Proteins in Potato

In total, 190 putative bHLH transcription factors were identified by HMMER [59]. Then, the bHLH protein sequences encoded by non-representative transcripts were excluded. The remaining sequences were checked for the existence of complete bHLH domains using CDD, Pfam and SMART. In total, 124 sequences were confirmed as potato bHLH proteins. Based on their chromosomal locations, the proteins were assigned from StbHLH1 to StbHLH124 (Table S3). The number of potato bHLH proteins was similar to that of tomato, a sibling species of potato belonging to *Solanaceae*, in which 159 bHLH proteins were identified [36]. The length of StbHLH proteins varied from 62 (StbHLH67) to 708 (StbHLH12) amino acids. Generally, the conserved bHLH domain contains ~60 amino acids [33]. The divergent lengths of potato bHLH proteins were mainly the result of other domains not the bHLH domain. The molecular weights ranged from 7.5 kDa (StbHLH67) to 75.9 kDa (StbHLH92). *StbHLH* genes were randomly distributed on 12 chromosomes, while three genes (*StbHLH1*, *StbHLH2* and *StbHLH3*) could not be anchored on any of the potato chromosomes. Only 623 Mb (86%) of the assembled genome are genetically anchored, and the constructed pseudomolecules for the 12 potato chromosomes harbor 90.3% of the predicted genes [55], which means 9.7% of the predicted genes could not be anchored on any of the 12 chromosomes. The predicted isoelectric point values of StbHLH proteins were between 4.66 (StbHLH51) and 10.42 (StbHLH67).

### 3.2. Gene Structure and Motif Analysis of StbHLH

**Figure 1:**
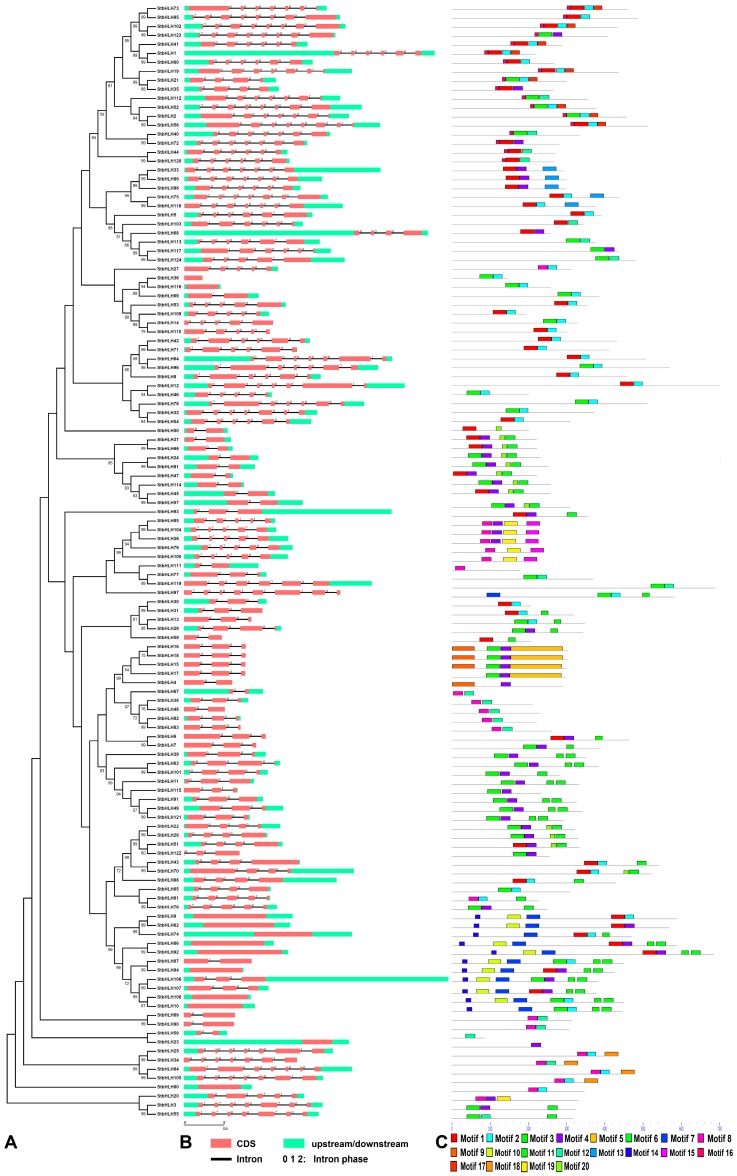
Gene structures of *Solanum tuberosum* basic/helix–loop–helix (*StbHLH*) genes and phylogenetic relationships, conserved motifs of StbHLH proteins. (**A**) Phylogenetic tree of 124 StbHLH proteins. The unrooted Neighbor-joining phylogenetic tree was constructed with MEGA6 [61] software using the full-length amino acid sequences of 124 StbHLH proteins. (**B**) Exon–intron organization of *StbHLH* genes. Red boxes represent exons and black lines of the same length represent introns. The upstream and downstream regions of *StbHLH* genes are indicated by green boxes. The numbers 0, 1 and 2 represent the intron phases. The sizes of exons can be estimated by the scale at bottom. (**C**) Arrangements of conserved motifs in the StbHLH proteins. Twenty predicted motifs are represented by different colored boxes, and motif sizes are indicated by the scale at bottom. For motif details refer to Table 1. CDS: coding sequence.

Gene organization plays a vital role in the evolution of multiple gene families [60]. A Neighbor-joining phylogenetic tree was constructed with MEGA6 [61] (Figure 1A). The genomic sequence and corresponding cDNA sequence of the same *StbHLH* gene were submitted to GSDS [48] together to show its gene structure. The number of introns varied from zero to seven (Table S3, Figure 1B). In addition, 12 (9.7%) genes were intronless, while 19 (15.3%) genes contained one intron. The remaining genes had two or more introns. Genes with few or no introns are considered to show lower expression levels in plants, which is different from that in animals [62]. However, the compact gene structure may contribute to the rapid expression of genes in response to endogenous and/or exogenous stimuli [63]. For example, intronless genes, *StbHLH65 and StbHLH76*, were expressed with low levels in most tissues, but were upregulated in response to heat stress. Owing to the closely related duplication relationship, the tandem duplicated genes contained the same amounts of introns. For example, the tandem duplicated genes *StbHLH15*, *StbHLH16*, *StbHLH17* and *StbHLH18* have two introns. Three intron phases are generally acknowledged, phases zero, one and two, in which the splicing occurred after the third, first, and second nucleotide, respectively [64]. Also, a conserved intron phase was observed through the *StbHLH* gene family (Figure 1B). The number of introns with intron phases of zero was 351, which accounted for 86.2% of the introns.

The MEME [46] program was used to identify the conserved motifs of StbHLH proteins. Meanwhile, the predicted motifs were annotated. Twenty conserved motifs were identified and varied from 10 to 138 residues in length (Figure 1C). Details of the 20 motifs were listed in Table 1. Each StbHLH protein contained different numbers of conserved motifs, ranging from one to seven. StbHLH23, StbHLH59 and StbHLH111 only had one conserved motif, while seven StbHLH proteins each contained seven conserved motifs. All of the predicted motifs were identified only once in each StbHLH protein sequence. In general, closely related StbHLH proteins on adjacent clades of the phylogenetic tree had the same or similar motif structures. For example, StbHLH33, 99, 98, 75 and 118 each contained two motifs, one and 13. The strong sequence diversity outside of the bHLH domain suggested that the bHLH family experienced extensive domain shuffling after the genome duplication [10]. In our research, 20 different motifs in various arrangements were found among potato bHLH family members. Thus, broad domain shuffling occurred in the protein structures of the bHLH family members.

### 3.3. Phylogenetic Analysis of the StbHLH Protein Family

**Figure 2:**
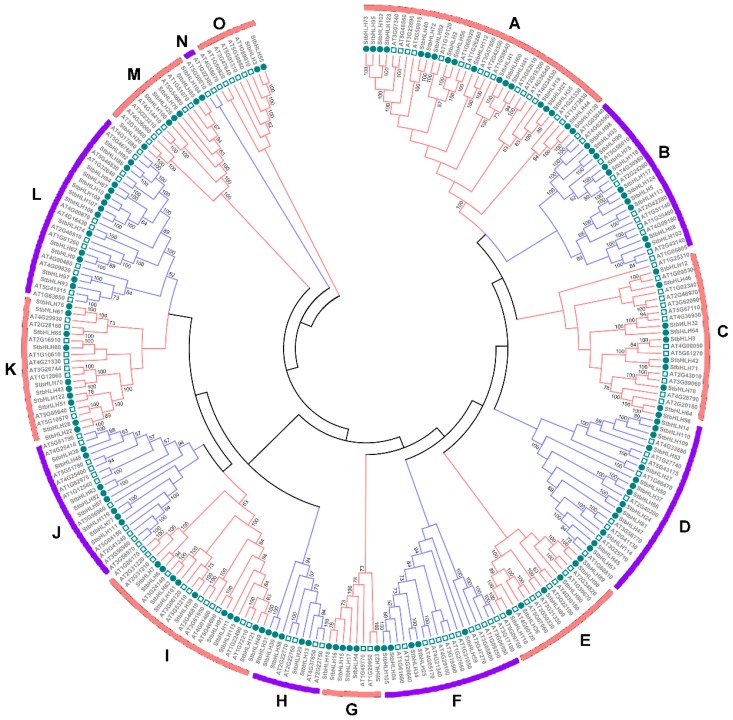
Phylogenetic tree of *Arabidopsis* and potato bHLH proteins. The phylogenetic tree was constructed using the Neighbor-joining method with 1000 bootstrap replications. The 15 subfamilies are marked with different colors. The circles represent potato bHLH proteins, and the rectangles represents *Arabidopsis* bHLH proteins.

To analyze the evolutionary relationships among the StbHLH members, 124 StbHLH proteins were aligned with 135 bHLH proteins from *Arabidopsis* [28] using ClustalX 1.83 [65]. An unrooted phylogenetic tree was constructed by MEGA6. In total, 259 bHLH protein sequences were classified into 15 distinct groups, which were named from A to O (Figure 2). The result corroborated the previously proposed classification of the bHLH family [27,66]. Group A was the largest subfamily with 34 proteins, while the smallest, Group N, contained only two proteins, both bHLH proteins (AT1G22380 and AT5G50010) were from *Arabidopsis*. StbHLH proteins were more closely related to those in the same subfamily from *Arabidopsis* than with the other bHLH proteins from potato. Most StbHLH proteins in the same group shared consensus motifs, and similar exon–intron structures within the corresponding genes (Table S4). For instance, 10 *StbHLH* genes in Group I contained two or three introns, with four genes having two introns and the others having three. Genes with the same number of introns only varied in lengths of exon and non-coding regions. Additionally, the 10 proteins in Group I shared the same motif arrangement of motifs three, four and six, except for StbHLH39 and StbHLH121, which lacked motif 6, and in StbHLH49, which had motif 1 instead of motif 3. A few of the clades of the phylogenetic tree were supported by low bootstrap values, which may be caused by relatively less informative character positions outside of the short and highly conserved bHLH domains [33].

### 3.4. Chromosomal Location and Gene Duplication

**Figure 3:**
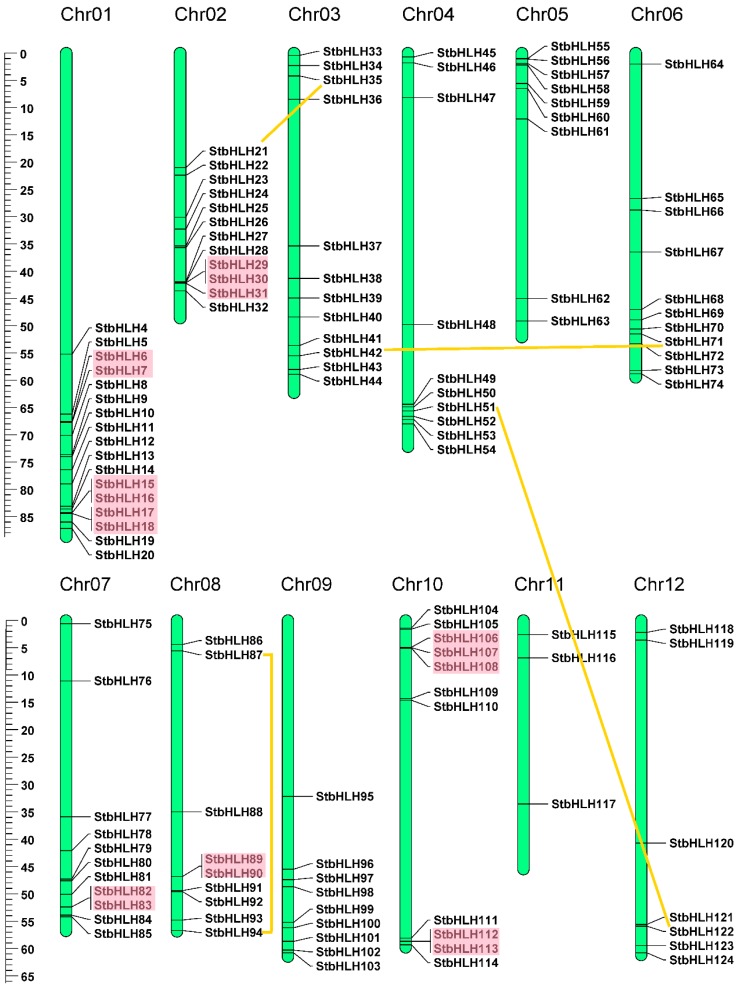
Chromosomal distribution and gene duplications of *StbHLHs*. The tandem duplicated genes are represented by red rectangles, and the segmental duplicated genes are linked by orange lines. The scale bar on the left indicated the length (Mb) of potato chromosomes.

The distribution of *StbHLH* genes was uneven on the chromosomes (Figure 3). The number of *StbHLH* genes on each chromosome varied from three to 17. Chromosome 1 contained the largest number, 17, of *StbHLH* genes. Chromosome 11 had only three *StbHLH* genes. The majority of *StbHLH* genes were located on the proximate or the distal ends of potato chromosomes. The detailed chromosomal locations of *StbHLH* genes are shown in Table S1. Additionally, the lengths of chromosomes can be estimated by the scale on the left. The results were consistent with those of tomato [36].

Genome duplication events occurred during the process of plant evolution [67,68]. The expansion of gene families and genomic evolutionary mechanisms mainly depend on gene duplication events [69]. The major duplication patterns are tandem and segmental duplication [70]. Gene duplication events were also identified for *StbHLH* genes. According to the defined criteria, 20 (16.1%) *StbHLH* genes were confirmed to be tandem duplicated genes. In addition, four pairs of *StbHLH* genes were found to be segmental duplicates. The 20 tandem duplicated genes formed eight gene clusters that were indicated by red blocks in Figure 3. Paralogs of segmental duplications were linked to each other by orange lines. A total of 28 (22.6%) *StbHLHs* were duplicated genes, which indicated that gene duplication played an important role in the expansion of *StbHLH* family.

### 3.5. GO Annotation of StbHLH Proteins

**Figure 4:**
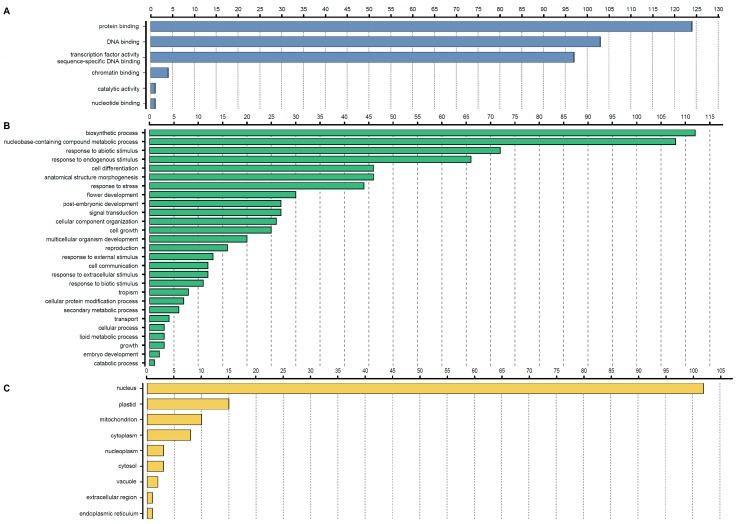
Gene ontology (GO) annotation of StbHLH proteins. The annotation was performed on three categories, (**A**) molecular function, (**B**) biological process and (**C**) cellular component.

The highly divergent sequences outside of the conserved bHLH domain indicate that bHLH proteins are involved in multiple biological processes. In *Arabidopsis*, a number of bHLH proteins were functionally characterized [71,72,73,74,75]. Thus, to understand the biological processes associated with *StbHLH* genes, we used *Arabidopsis* as the reference species to perform a GO annotation of potato bHLH proteins. The results are shown in Figure 4 and Table S5. The majority of StbHLH proteins were involved in protein and DNA binding. Meanwhile, four proteins (StbHLH43, StbHLH70, StbHLH88 and StbHLH122) functioned in chromatin binding. In addition, StbHLH119 was involved in both catalytic activity and nucleotide binding. Most of the StbHLH proteins (102, 82.3%) were located on the nucleus. However, there were also StbHLH proteins located on plastids (15) and mitochondrion (10), and in the cytoplasm (8). A small number of StbHLH proteins were located on the nucleoplasm (StbHLH8, 42, 71), cytosol (StbHLH68, 103, 117), vacuoles (StbHLH59 and 119), extracellular regions (StbHLH119) and the endoplasmic reticulum (StbHLH119). Additionally, some StbHLH proteins existed on multiple cellular components. For example, StbHLH119 was located on three cellular components, vacuoles, extracellular regions and the endoplasmic reticulum, which may reflect its multifunction in various biological processes. The biological process analysis showed that StbHLH proteins participated in various biological processes. The biosynthetic process and nucleobase-containing compounds’ metabolic processes involved the greatest number of StbHLH proteins. Besides, StbHLH proteins could respond to abiotic stress. Also, there were ~25–45 StbHLH proteins involved in post-embryonic development, flower development, anatomical structure morphogenesis and cell differentiation. Thus, based on the biological process analysis, StbHLH proteins could function in multiple biosynthetic and metabolic processes, in response to abiotic and biotic stresses, in the development of various tissues and organs, in signal transduction and in cell communication.

### 3.6. The StbHLH Expression Pattern in Various Tissues

**Figure 5:**
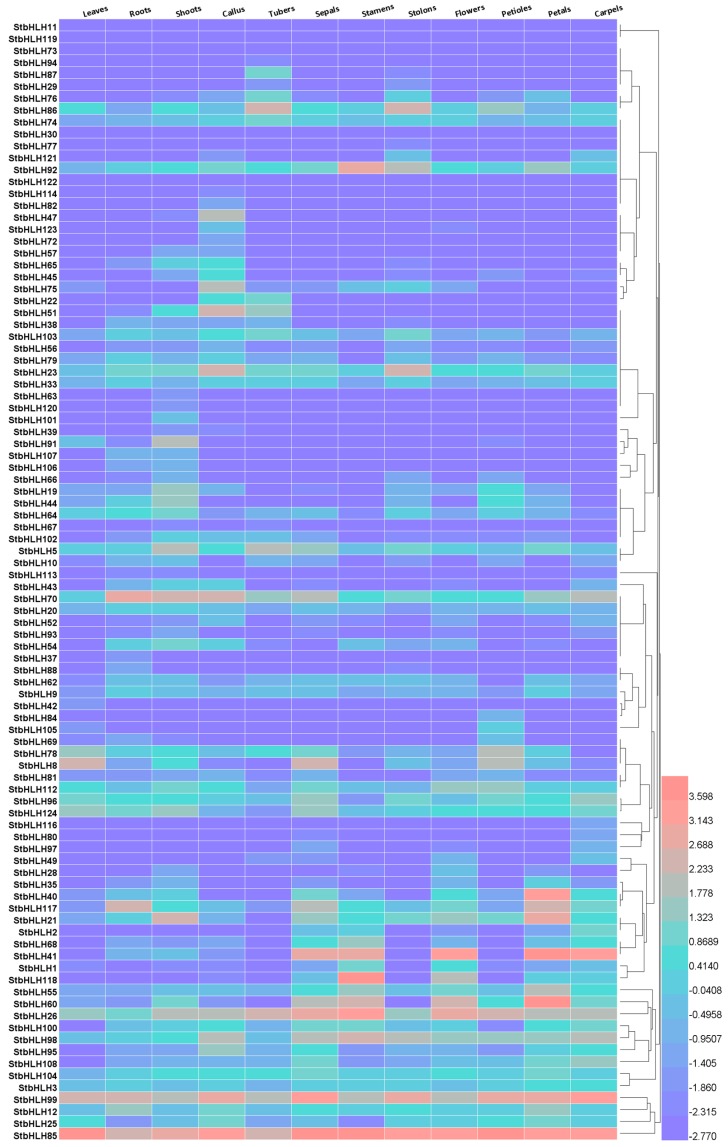
Expression heatmap of *StbHLH* genes in different tissues and organs. Fragments per kilobase of transcript per million mapped reads (FPKM) values of *StbHLH* genes were transformed by log2, and the heatmap was constructed with HemI. The clustering tree was constructed by hierarchical clustering using the average linkage method.

To dissect the expression patterns of *StbHLH* genes in various tissues, RNA-seq data, represented by fragments per kilobase of transcript per million mapped reads (FPKM, Table S6), was downloaded from PGSC. We processed the FPKM data and generated a heatmap. Twelve different tissues and organs were included in the analysis. The *StbHLH* genes with FPKM values less than two in all 12 tissues were considered to be barely expressed. Thus, 30 unexpressed genes were excluded from the heatmap (Figure 5). The remaining 94 *StbHLH* genes used in the heatmap were expressed in at least one tissue. Two genes (*StbHLH85* and *StbHLH99*) showed high expression levels across all of the analyzed tissues. Some genes have been identified as highly expressed in various tissues [76]. These highly expressed genes may possess specific housekeeping activities. Additionally, some genes were expressed in most of the analyzed tissues, and some gene expressions were tissue specific. *StbHLH82*, *47*, *123* and *72* were expressed only in the callus. *StbHLH76* and *StbHLH86* had a relatively high expression level in the tuber and stolon compared with in other tissues. This suggests that the two genes may be involved in tuber and stolon development. *StbHLH68*, *41*, *1*, *118*, *55*, *60* and *26* were highly expressed in flower tissues, including the sepal, stamen, flower and petal. These genes may be related to flower development. In addition, *StbHLH1*, *41* and *60* were also included in flower development by the GO analysis (Figure 4, Table S5), which further suggested our conclusion.

**Figure 6:**
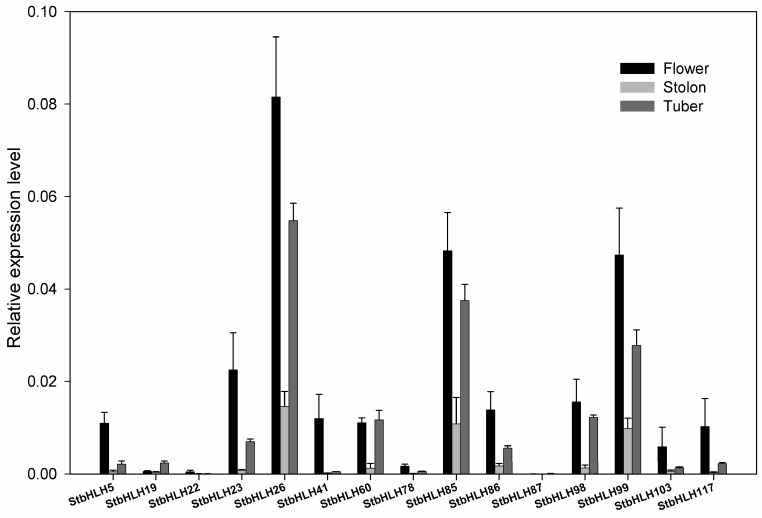
Expression profiles of *StbHLH* genes in various tissues. Quantitative RT-PCR was used to investigate the expression levels of *StbHLH* genes, and the results are represented by means ± standard deviations. The relative expression level was calculated by 2^−ΔCT^ method comparing with that of *ef1α.*

To further examine the expression patterns of *StbHLH* in different tissues, we used qRT-PCR to quantify the expression levels of several *StbHLH* genes in *Desiree*. *Desiree* is a tetraploid that is similar to cultivars in its genetic background. Three tissues, flower, stolon and tuber, were used for qRT-PCR analysis. StbHLH117, with low expression in the three tissues, and 14 StbHLHs, with high expression in at least one of the three tissues, were selected for analysis (Figure 6). Three *StbHLHs* (*StbHLH26*, *85* and *99*) had relatively higher expression levels in the three tissues than other *StbHLHs*. Three genes (*StbHLH60*, *85* and *98*) expressed similar abundance levels between flower and tuber. *StbHLH19* was observed with a higher expression level than in the other two tissues, while *StbHLH41* was mainly expressed in the flower. The expression of *StbHLH87* could be barely detected. However, based on qRT-PCR, only three genes (*StbHLH26*, *41* and *78*) had the same expression patterns as determined by the RNA-seq data. The differences in expression patterns between the qRT-PCR and heatmap in various tissues may have been caused by the vastly divergent genetic backgrounds of the two varieties.

Based on the phylogenetic tree, *StbHLH22* and *AtSACL3* (AT1G29950) were orthologs. *AtSACL3* is a member of a negative feedback loop which contributes to the maintaining of root apical meristem size and proper root growth [77]. Thus, *StbHLH22* may function in the same way in potato, and highly express in the root. In the same way, *StbHLH78* was found to be an ortholog of *AtPIF4* (AT2G43010) and *AtPIF5* (AT3G59060). The *AtPIF* genes are the central signaling hub that functions in regulating plant growth and development [78]. *AtPIF4* is also found to contribute to hybrid vigor through the auxin pathway [79]. *StbHLH78* had a relatively high expression in the leaf, petiole (Figure 5) and flower (Figure 6). It may be inferred that *StbHLH78* has the same function in regulating potato growth and development.

### 3.7. Expression Analysis of StbHLH under Abiotic Stresses

**Figure 7:**
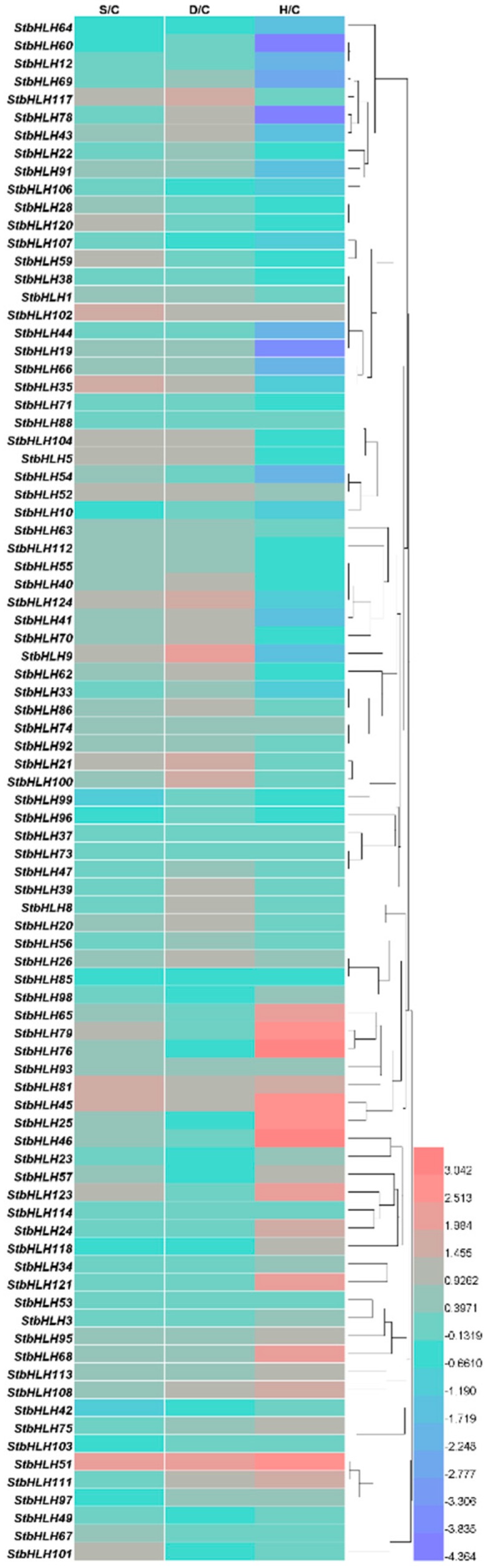
Expression heatmap of *StbHLH* genes under salt, drought and heat stress. FPKM values of *StbHLH* genes were transformed by log2, and the heatmap was constructed with HemI. The clustering tree was constructed by hierarchical clustering using average linkage method.

To analyze the stress responses of *StbHLH* genes, we compared the FPKM values (Table S7) of *StbHLH* genes under heat, salt and drought stress with those of the corresponding controls. A total of 38 *StbHLH* genes had FPKM values lower than two under the three stress treatments and in the corresponding controls, and were, therefore, excluded from the heatmap (Figure 7). Based on the heatmap, we concluded that a number of *StbHLH* genes respond to abiotic stresses, including salt, drought and heat. Under heat stress, several *StbHLH* genes were extremely up-regulated, such as genes *StbHLH65*, *76* and *79*, or downregulated, such as genes *StbHLH19*, *60* and *78*. However, the variations in the *StbHLH* genes’ expression levels under salt and drought stress were not as divergent. In addition, a few *StbHLH* genes responded to all three stresses. For instance, *StbHLH51* was sensitive to salt, drought and heat stress, and was up-regulated distinctly under the three stresses. In addition, several *StbHLH* genes, such as *StbHLH69* and *StbHLH78*, showed contrasting expression patterns among the three stresses. *StbHLH78* was not sensitive to salt stress, but it was up-regulated under drought stress and down-regulated under heat stress. One single bHLH protein could interact with one or more bHLH proteins and even non-bHLH proteins [3]. The *StbHLH* genes with different stress responses may form heterodimers with specific bHLH proteins, resulting in diverse stress responses and expression patterns.

**Figure 8:**
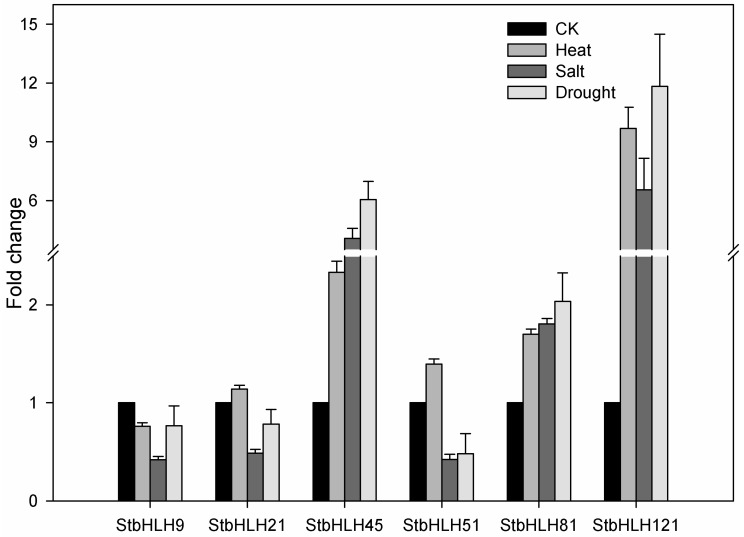
Expression levels of *StbHLH* genes under heat, salt and drought stress. Quantitative RT-PCR was used to investigate the expression levels of *StbHLH* genes, and the results are represented by means ± standard deviations. The relative expression levels of *StbHLH* genes under different abiotic stresses are compared with that of the control (CK).

Moreover, three *StbHLHs* (*StbHLH45*, *51* and *81*) which were upregulated under three abiotic stresses and three *StbHLHs* (*StbHLH9*, *21* and *121*) which were in response to drought or heat stress were analyzed for their expression profiles (Figure 8). Of the six *StbHLH* genes, *StbHLH45*, *StbHLH81* and *StbHLH121* were more sensitive to the three abiotic stresses than the other three *StbHLH* genes and were notably upregulated (>2-fold) under the three abiotic stresses. In comparison, *StbHLH9*, *StbHLH21* and *StbHLH51* showed different expression patterns within the three abiotic stresses. Additionally, we compared the results of qRT-PCR with the heatmap. It was confirmed that two genes (*StbHLH45* and *StbHLH81*) had the same expression patterns under the three abiotic stresses. For the other genes, the same expression levels were observed under one or two abiotic stresses. The differences between the two sets of results acquired by qRT-PCR and RNA-seq may be caused by various factors. Although the same potato variety was used, only the aboveground part of the plantlet was sampled in our research, while the whole plant was collected for RNA-seq. Under heat stress, the plantlets were treated for 24-h under a normal photoperiod (16 h light/8 h dark) in our study, but they were treated in darkness all the time for RNA-seq. The potato RNA-seq data downloaded from PGSC was presented as FPKM values. Compared with raw read counts, FPKM values can better reduce sample differences. However, there are also deficiencies in the FPKM data. For example, some highly expressed genes could alter the FPKM values [80]. The bias in FPKM values can lead to different expression patterns compared with qRT-PCR data. The orthologs of six *StbHLHs* have not been functionally characterized in *Arabidopsis* except for *AtABS5* (AT1G68810), which is an ortholog of *StbHLH45*. In Arabidopsis, *AtABS5* are involved in vascular cell division in root apical meristem [81] and leaf morphogenesis [25]. It may be inferred that *StbHLH45* functions in the regulation of potato growth and development. The sensitive response to the abiotic stress of *StbHLH45* may reflect its role in stress tolerance, which needs to be further analyzed.

## 4. Conclusions

In summary, we performed a genome-wide analysis of the bHLH family in potato, and 124 *StbHLH* genes were identified. The divergent biochemical characteristics of StbHLH proteins were analyzed. Based on the phylogenetic tree, StbHLH proteins were classified into 14 subfamilies, named from A to O, except for group N that did not have a StbHLH protein member. The similar exon–intron structures of the genes and motif arrangement of the StbHLH proteins within the subfamilies further supported the classification predicted by the phylogenetic tree. *StbHLH* genes were distributed on 12 potato chromosomes. A total of 28 *StbHLH* genes were confirmed to be duplicated genes, which indicated the important role of gene duplication in the expansion of the *StbHLH* family. The GO analysis revealed multiple functions for the StbHLH proteins. The RNA-seq and qRT-PCR illustrated that *StbHLH* genes were expressed in various tissues and responded to different abiotic stresses on the transcriptional level. The results provide comprehensive information for further functional analyses of the *StbHLH* gene family.

## Figures and Tables

**Table 1:** Sequences of 20 predicted motifs of StbHLH proteins.

Motif	Width	Motif Sequence	Annotation
1	36	[RK]RGQA[TA]D[SP]H[SV][LE]AER[RK]RRE[KR][IL][NS][EQ]R[MLF][KY]AL[QR][DS][LV]VP[NG]C[NS]K	Helix–loop–helix DNA–binding domain
2	24	[TM][DG]KASML[DG][ED][AI]I[EN]Y[VIL][KQ][FSQ]LQLQ[VI][KEQ]FL	Helix–loop–helix DNA–binding domain
3	36	KR[EG]S[AQ][RT]XXH[SI]LAERRRR[EK][KR][ILM][NS]ER[LFM]XAL[RQ][SE]LVP[NG]STK	Helix–loop–helix DNA–binding domain
4	26	[MT]DKAS[IL]L[GD][DE]A[IV][DN][YH][VI]KEL[KQ]X[QK]VQ[EK]L[ES]S	Helix–loop–helix DNA–binding domain
5	138	QKL[EK]RL[EK]EYSI[RK]LM[SG]SQK[VI]GNSWEKY[VL]GDQGST[NC]NST[AT]ITP[TI][TN]HGASPLIP[TK][GS]FMTWSS[PL]NVILN[IV]CGEDAHISVCCPKKPGLFT[IM]ICYVLEKH[KN]I[DN]IV[SF]AQISSDQFRSMFMIQAHAKG[GE][SR][GE][VIL][AT]QFS[GV]AF[TK]VE[DE][MR][YL]K	–
6	26	[KRS][LT][MI]X[AT]L[QEK]SLGLD[VI]LHA[NS][IV][ST][TS][VL][GN][GD][LRF][VM]L	signal peptide
7	36	[HA]GIQT[IFL]VCIPTS[NS]GV[VL]ELGS[STV][EQ][LV]I[KP][EQ][DNS]L[EN]L[VI]QQ[VI]KS	bHLH–MYC N–terminal
8	26	D[RA]E[KR][LQ]RREK[LM][NS][DE][RKL][FIY]QEL[RQ]SL[LV]PPGR[KP]	Helix–loop–helix DNA–binding domain
9	59	MNG[GS]GENN[HD][GV][LF]PW[EG]TND[FLV]WSYLNLND[IN]Q[IV]GS[GE][EV]TFEGDKLPD[PL]TRSDT[CY]QPLTV[VI]NEV[VI]	–
10	34	E[WM]F[YF]L[MAIV]S[ML][APTY][QF][SC]F[SVP][NRV][GE][DE]G[LVG][PV]GK[AC][FY][SY]S[GDS][SK][HFP][VI]W[LV][TAS][GD][ADTY]	bHLH–MYC N–terminal
11	19	[ED][IV][ED]V[KR]I[IV][GE]X[DE][AV][ML][IVL][RK][IV]Q[SC]E[KRN]	–
12	26	[RTM][SDGN]T[AS][DS][MVH]L[DQ]E[AIT][VI][NE]Y[IV][KQ]SL[QK]N[QN][VI][EK][EF]L[SE][KM]	Helix–loop–helix DNA–binding domain
13	36	E[HQR][QE]VAKLMEE[DN][VM]G[AST]AMQ[YF]LQ[SG]K[GSA]LC[IL]MP[IV]SLA[ATS][AL]I[YS]	–
14	14	AX[ES][SDW]WAYAIFWQSS	–
15	35	K[LM][MVA][PV][FIY][ILMP][SG]Y[PG][GSY][VI][AP]MWQ[FY][MLV][PQ]P[AS][ASV][VIR]DTS[QE]DH[VMS]LRPP[VA]A	–
16	10	[PK][PK]KDY[IV]HVRA	–
17	23	SMKL[AE][TA]VNPR[LM][DN]F[DN]I[DE][ANS][LI][LFP][AS]K[DE][IFM]	–
18	36	[DG]LRS[RK]GLCLVP[IV]SSTFP[VL][AT][HAT]ET[ANST][VMT][DE][FL]WTP[TN][FL]G[GRS]TFR	–
19	36	LQE[KE]IKELK[AV]EKNELR[DE]EKQRLK[AS][ED]KEKLEQQLK[AT][MT]	–
20	14	[ITV]K[AI][SE][IL]CC[ED]D[RK][PS][EGD]LL	–

– Means no annotation was found. The square brackets indicated all the possible amino acids at the site.

## Supplementary Materials

The following are available online at http://www.mdpi.com/2073-4425/9/1/54/s1. Table S1: Gene IDs and names of *Arabidopsis* bHLH proteins. Table S2: Primer sequences used in qRT-PCR. Table S3: Features of *StbHLHs* identified in potato. Table S4: Number of *StbHLHs* with different intron numbers in each phylogenetic group. Table S5: Protein IDs in each category of GO annotation. Table S6: FPKM values of *StbHLH* genes in various tissues. Table S7: FPKM values of *StbHLH* genes under salt, drought and heat stress.
